# Relationship between Circadian System Status, Child–Pugh Score, and Clinical Outcome in Cirrhotic Patients on Waiting Lists for Liver Transplantation

**DOI:** 10.3390/jcm13154529

**Published:** 2024-08-02

**Authors:** Laura Martínez-Alarcón, Antonio Martínez-Nicolás, Marta Jover-Aguilar, Víctor López-López, Felipe Alconchel-Gago, Antonio Ríos, Juan Antonio Madrid, María de los Ángeles Rol, Pablo Ramírez, Guillermo Ramis

**Affiliations:** 1 Departamento de Producción Animal, Hospital Clínico Universitario Virgen de la Arrixaca (UDICA), 30120 Murcia, Spain; lma5@um.es; 2 Instituto Murciano de Investigación Biosanitaria (IMIB), 30120 Murcia, Spain; antilas@um.es (A.M.-N.); marta.jover@um.es (M.J.-A.); jamadrid@um.es (J.A.M.); angerol@um.es (M.d.l.Á.R.); 3 Chronobiology Laboratory, Department of Physiology, College of Biology, University of Murcia, Mare Nostrum Campus, 30100 Murcia, Spain; 4 Human Physiology Area, Faculty of Sport Sciences, University of Murcia, Santiago de la Ribera-San Javier, 30720 Murcia, Spain; 5 Ciber Fragilidad y Envejecimiento Saludable (CIBERFES), 28029 Madrid, Spain; 6 Servicio de Cirugía, Hospital Clínico Universitario Virgen de la Arrixaca, 30120 Murcia, Spain; victorrelopez@gmail.com (V.L.-L.); alconchelgago@gmail.com (F.A.-G.); arzrios@um.es (A.R.); ramirezp@um.es (P.R.); 7 Departamento de Producción Animal, Facultad de Veterinaria, Campus de Espinardo, Universidad de Murcia, 30100 Murcia, Spain

**Keywords:** cirrhosis, distal temperature, physical activity, hematology, biochemistry

## Abstract

**Background/Objectives:** Many patients suffering from liver cirrhosis are eventually added to waiting lists for liver transplantation whose priority is established based on scales such as the Child–Pugh score. However, two marker rhythms of the circadian system, motor activity and distal temperature, are not evaluated. **Methods:** To determine the relationship between the functional status of the circadian system and the Child–Pugh scale in patients awaiting liver transplantation, distal temperature, motor activity, and light exposure rhythms were monitored for a full week using a wrist device (Kronowise 6.0) in 63 patients (17 women, 46 men) aged between 20 and 76 years. **Results:** Circadian parameters (amplitude, regularity, and fragmentation) of motor activity rhythms, distal temperature, and light exposure worsen in close association with liver disease severity as assessed by using the Child–Pugh score. Likewise, the worsening of rhythmic parameters and liver disease is associated with a deterioration in the markers of the red series: count, hemoglobin, and hematocrit. **Conclusions:** These results indicate the utility of ambulatory monitoring of marker rhythms to complement the clinical information provided by the Child–Pugh scale and to help establish nutrition, physical exercise, and sleep guidelines that promote better survival and quality of life in these patients.

## 1. Introduction

Liver cirrhosis (LC) is a worldwide health concern and often results in liver transplantation. In addition, it is associated with several disorders such as sarcopenia, hepatic encephalopathy, malnutrition, frailty, severe cognitive impairment [1,2,3], and sleep disorders [4,5,6,7,8,9]. Chronic liver patients suffer a progressive loss of quality of life, which often leads to a slower recovery or even hinders the viability of liver transplants (quality of life after liver transplantation) [10]. In addition, LC is a cause of recurrent hospital admission, especially in those patients who develop hepatic encephalopathy [9]. Hepatic impairment leads to the disturbance of several metabolic pathways, with alterations in muscle synthesis, and the coagulation and metabolism of carbohydrates, fats, and proteins, which can lead to severe malnutrition and significantly worsen the prognosis of patients [7]. All of this results in analytical alterations that allow for the diagnosis and prognosis of patients, with alterations in liver enzymes, coagulation factors, coagulation indicators, or the red and white blood series [7,8].

In the context of personalized medicine, the care of people with LC through improvements in physical exercise, sleep, and nutrition constitutes essential factors. Undoubtedly, the care of liver patients on the transplant waiting list must take a multidisciplinary approach that includes a nutrition consultation, rehabilitation, physiotherapy, and sleep disorder consultations, if necessary. A holistic approach to these patients could improve their state of health, beyond their liver disease, at the time of transplantation and allow the patient to make a faster and more successful recovery.

LC patients suffer with sleep disorders, reaching an incidence of 50–65%. The most frequent sleep disorders are insomnia, high sleep fragmentation, excessive daytime sleepiness, and delayed sleep phase [4,5,6,7,8,9]. These sleep disorders are associated with a delay in the secretion patterns of two of the most used marker rhythms of the circadian system, melatonin and cortisol [7,11,12]. The assessment of the secretion pattern of melatonin and cortisol or the core body temperature rhythm (another frequently used circadian marker rhythm) requires the active participation of the patients, disturbing their night sleep. However, alternative marker rhythms such as motor activity or distal skin temperature rhythms using wearable devices do not require the collaboration of the patient [13,14,15,16]. Distal skin temperature was proposed as the most practical and least invasive measure to evaluate the circadian phase by a consensus document, “Developing Biomarker Arrays Predicting Sleep and Circadian-Coupled Risks to Health”, sponsored by the National Heart Lung and Blood Institute, the National Institute on Aging, and the Sleep Research Society [12,15,17,18]. This statement is based on its strong endogenous component [19,20,21,22,23,24], ease of monitoring [25,26,27,28], ability to assess the circadian phase [20,23], and validation against polysomnography [15,29].

This study aimed to assess the relationship between the circadian system status (evaluated through ambulatory monitoring of motor activity, distal skin temperature, and light exposure) and the Child–Pugh score used to assess the chronic liver disease and cirrhosis of patients on transplant waiting lists. This information can be useful for disposing of additional criteria to stratify the immediacy for transplantation and to design personalized intervention strategies aimed at improving the quality of life and the success of liver transplantation.

## 2. Materials and Methods

### 2.1. Patients

A sample of 63 patients, comprising 17 women and 46 men, with a mean age of 57.9 ± 9.3 (from 20 to 76 years old; males = 57.7 ± 9.7; females = 58.4 ± 8.5), was taken from the transplant waiting list of Hospital Clínico Universitario Virgen de la Arrixaca and classified as Child A, Child B, and Child C, as previously described [30,31]. The patients included in this study were all patients on the waiting list for transplantation during the years 2021 and 2022 who were recruited for the PI20/00981 CRONORGAN 21 project funded by the Ministry of Economy and Competitiveness and the Instituto de Salud Carlos III. The inclusion criteria were as follows: a. being on the waiting list for liver transplantation; b. patients who offered sufficient guarantees of adherence to the protocol; c. patients who gave written informed consent to participate in this study; d. patients over 18 years of age. The exclusion criteria were as follows: a. patients with liver retransplantation; b. patients with an inability to fully understand the informed consent; c. patients who did not give their written informed consent to participate in this study; d. patients who did not offer sufficient guarantees of adherence to the protocol. Patients were also eliminated if they did not wear the actigraph for at least one week, regardless of the cause, as this was necessary to obtain robust data on temperature, movement, and light received. The characteristics of the population studied are listed in Table 1.

There was no difference between the observed frequencies for both sexes and the expected frequency (*p* = 0.399). There were also no significant differences when biometric parameters were compared, either taken together or divided according to sex. Patients were informed of this study on the same day as their inclusion on the transplant waiting list. They were informed that withdrawal or noncompliance would have no consequences on their therapeutic approach and signed the informed consent before the study launch. This study follows the bioethical principles set out by the Declaration of Helsinki. The data from the volunteers were protected according to Spanish Law 15/1999 from 13 September. This study was approved by the Hospital Clínico Universitario Virgen de la Arrixaca Ethics Committee.

### 2.2. Ambulatory Circadian Monitoring (ACM)

Ambulatory circadian monitoring was performed using a Kronowise 6.0^®^ (Kronohealth SL, Murcia, Spain), a watch-like device, placed on the non-dominant wrist for a whole week. This device comprises a temperature sensor (precision: ±0.1 °C, resolution: ±0.0635 °C, sample rate: 1 Hz) for distal skin temperature (DST, in degrees centigrade), a triaxial MEMS accelerometer (range: ±2 g, sensitivity: 0.001 g, sample rate: 10 Hz) for acceleration (in ∑g/30 s) and movement time (in counts per minute), and a lux meter (range: 0.01–43.000 lux, resolution: 16 bits, sample rate: 1 Hz) for visible light exposure (in Log_10_ lux), as previously described [32]. All of the recorded data were saved in 30 s epochs and downloaded from the data logger using Kronoware 10.0 (Kronohealth SL, Murcia, Spain).

### 2.3. Blood Parameters

At the recruitment stage, a blood sample was taken to determine routine blood parameters, both hematological and biochemical, including cell count, coagulation factors, metabolites, and liver enzymes. Biochemistry parameters include glucose (Glu), urea (Ure), sodium (Sod), creatinine (Crea), plasma proteins (PProt), albumin (Alb), total bilirubin (TBil), direct bilirubin (DirBil), alpha-fetoprotein (AFP), cholinesterase (Cholin), cholesterol (Chol), triglycerides (TG), HDL- and LDL-cholesterol (HDLC and LDLC), GOT-AST (aspartate aminotransferase), GPT-ALT (alanine aminotransferase), serum alkaline phosphatase (ALP), gamma-GT (GGT), transferrin (Trans), and ferritin (Ferr); hematological parameters include red blood cell count (RBC), hemoglobin (Hb), hematocrit (Hto), mean corpuscular volume (MCV), mean corpuscular hemoglobin (MCH), mean corpuscular hemoglobin concentration (MCHC), red cell distribution width (RDW), platelet count (PLT), mean platelet volume (MPV), platelet distribution width (PDW), white blood cell count (WBC), neutrophils (NEUs), lymphocytes (LYMPs), monocytes (MONOs), eosinophils (EOSIs), basophils (BASOs), fibrinogen (FIBRI), partial prothrombin time (PTT), activated partial thromboplastin time (APTT), international normalized ratio (INR), activated partial thromboplastin time (ATTP), and activated partial thromboplastin time ratio (APTTR).

### 2.4. Data Analysis

Wrong data produced by temporarily removing the sensors were eliminated when the rate of change concerning the previous value was higher than the interquartile distance (from Q1 to Q4), as previously reported [28]. The mean daily pattern for all variables was calculated per individual and then averaged per group (Child A, Child B, and Child C).

To characterize the circadian pattern of DST, acceleration, movement time, and light exposure, a non-parametrical analysis was performed. This analysis involved the calculation of interdaily stability (constancy of the 24 h rhythmic pattern over days, IS), intradaily variability (fragmentation of the rhythm, IV), relative amplitude (amplitude of the rhythm, RA), circadian function index (robustness of the rhythm, CFI), as previously reported [15,33], the mean of the variable (MEAN), and the midday value (mean of the 10 consecutive hours of higher values for variables with acrophase during the daytime (acceleration, movement time, and visible light) and lower values for variables with acrophase during the nighttime (DST), MIDDAY) and the midnight value (mean of the 5 consecutive hours of lower values for variables with acrophase during the daytime and higher values for variables with acrophase during the nighttime, MIDNIGHT) with their respective timings (TMIDDAY and TMIDNIGHT). RA was calculated as (MIDDAY − MIDNIGHT)/(MIDDAY + MIDNIGHT) for variables with the acrophase during the daytime, as described by [33], whereas it was calculated as (MIDNIGHT − MIDDAY)/(MIDDAY + MIDNIGHT) for DST, as previously reported [12].

### 2.5. Statistical Analysis

Data are expressed as mean ± SEM. Differences between groups were determined using the general linear model, controlling for age, gender, and BMI (body mass index), and the correlations between parameters were analyzed using a control for age and BMI partial correlation. A difference was determined as significant when *p* < 0.05 and a trend was considered significant when *p* < 0.1.

## 3. Results

### 3.1. Child–Pugh Score Influences Circadian Rhythm

The circadian patterns of all monitored variables for each Child–Pugh category (Child A, n = 18; Child B, n = 29; Child C, n = 16) are shown in Table 2.

**Figure 1:**
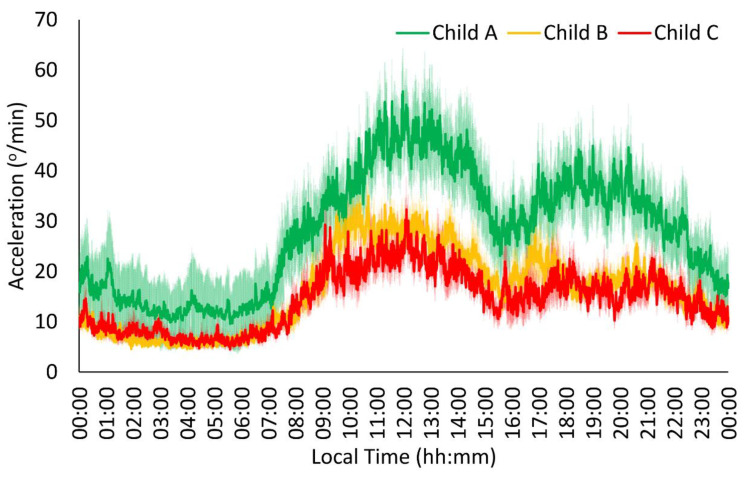
Acceleration pattern in patients with liver cirrhosis (n = 63). Mild patients or Child A (n = 19) are shown in green, moderate patients or Child B (n = 27) in orange, and severe patients or Child C (n = 17) in red. All data are expressed as mean ± SEM.

**Figure 2:**
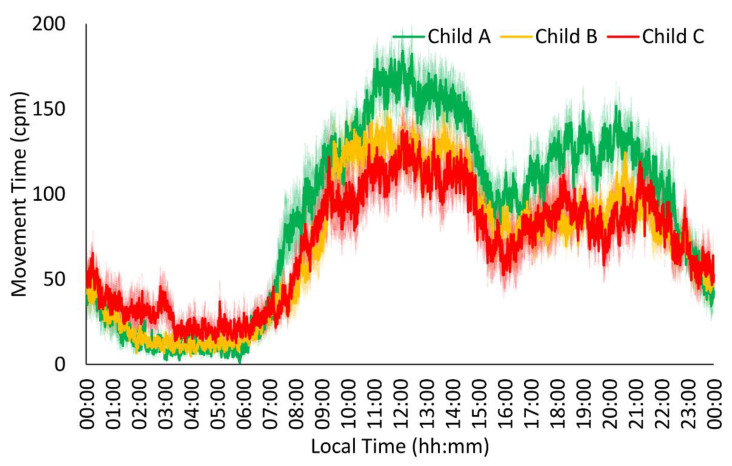
Time in movement rhythm in patients with liver cirrhosis (n = 63). Mild patients or Child A (n = 19) are shown in green, moderate patients or Child B (n = 27) in orange, and severe patients or Child C (n = 17) in red. All data are expressed as mean ± SEM.

All of the Child groups showed the typical acceleration and time in movement patterns (Figure 1 and Figure 2), exhibiting stable, low values at night (00:00 h–08:00 h) and variable, high values during the daytime (08:00 h–00:00 h), with a slight decrease in the postprandial time (15:00 h–17:00 h for Spanish schedules). The acceleration pattern of the Child C and Child B groups showed lower amplitude, robustness, midday, and mean values than the Child A group in the acceleration pattern (Table 2), together with a trend toward higher variability in the more severe groups (*p* = 0.059). For the time in movement, the Child C group exhibited lower stability, amplitude, robustness, midday, and mean values together with a trend toward higher variability compared to the Child A group (Table 2). In addition, the Child B group showed higher variability and lower midday values and mean values than the Child A group, whereas the Child C group exhibited lower amplitude and robustness values compared to the Child B group.

**Figure 3:**
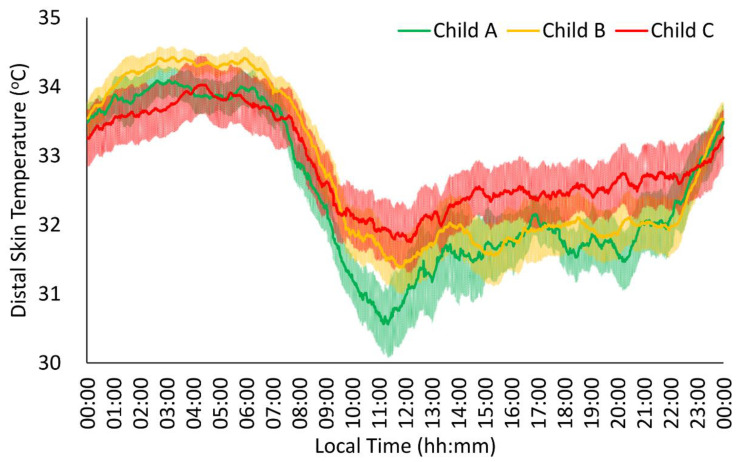
Distal skin temperature rhythm in patients with liver cirrhosis (n = 63). Mild patients or Child A (n = 19) are shown in green, moderate patients or Child B (n = 27) in orange, and severe patients or Child C (n = 17) in red. All data are expressed as mean ± SEM.

The distal peripheral temperature rhythm of all groups (Figure 3) presented the previously described characteristics of the pattern, with stable, high values at night (00:00 h–08:00 h) and variable, low values during the day (08:00 h–00:00 h). The Child C group showed lower stability, amplitude, and robustness values for distal skin temperature than the Child A group, together with a trend toward higher midday values (*p* = 0.064) and mean values (*p* = 0.056) in the more severe groups (Table 2).

**Figure 4:**
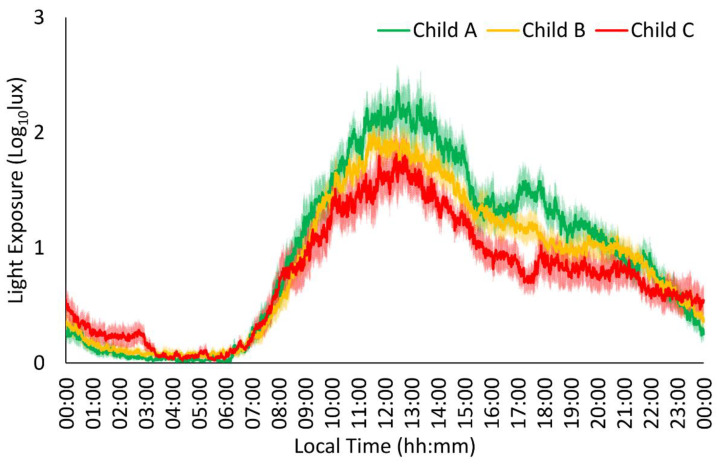
Light exposure pattern in patients with liver cirrhosis (n = 63). Mild patients or Child A (n = 19) are shown in green, moderate patients or Child B (n = 27) in orange, and severe patients or Child C (n = 17) in red. All data are expressed as mean ± SEM.

Light exposure in every group (Figure 4) showed stable, low values at night (00:00 h–08:00 h) and variable, high values during the day (08:00 h–00:00 h). In addition, the Child C group showed lower stability, robustness, and midday values than the Child A and Child B groups. Finally, the Child C group also exhibited lower mean values than the Child A group.

### 3.2. Biochemical Parameters Showed Alterations Related to Chronic Liver Disease

Blood parameters, depending on the Child–Pugh classification score, are listed in Table 3 and Table 4. Differences among groups were observed for sodium, HDLC, and GGT, with decreasing values as the Child–Pugh score increased.

Deviations from normal values were detected in glucose (increased), urea (increased), total and direct bilirubin (increased), and alpha-fetoprotein (decreased) in the Child A and C groups, and serum cholinesterase (decreased) in all groups; GOT increased in the Child A and C groups, GPT and ALP increased in all groups, GGT decreased in the Child B and C groups, transferrin decreased in the Child C group, and ferritin increased in the Child B and C groups.

Regarding hematological parameters, differences among the Child groups were found for RBC, Hb, Hto, WDP, and ACTP. The data are shown in Table 5.

Deviations from normal values were observed for RBC (decreased) in the Child B and C groups. Hb, PLT, and Hto decreased in all three groups; RDW increased in all groups, and the coagulation parameters PTT, APTT, and INR increased in all three Child groups.

As it seemed interesting to study the influence of hepatic encephalopathy, the parameters of the red series and PLT were analyzed, depending on the presence or absence of encephalopathy at the time of recruitment. The results are shown in Table 5.

There is a significant difference in all parameters depending on whether encephalopathy is present or not. Patients with encephalopathy have parameters that deviate from normal values, while patients without encephalopathy show slight deviations for RBC and PLT.

### 3.3. Correlations between Circadian Parameters and Blood Parameters

**Figure 5:**
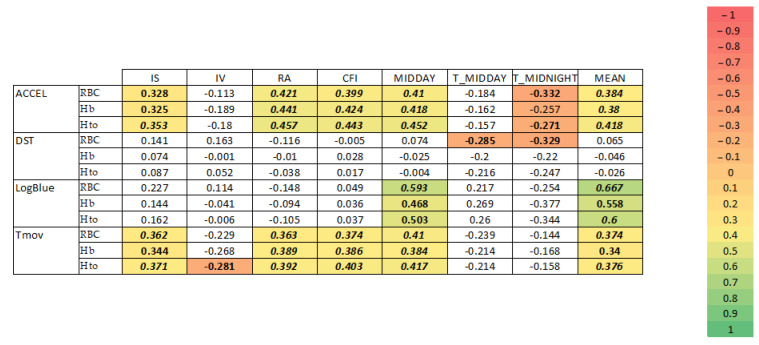
Controlled partial correlations for age and BMI. Correlations in bold type had significance level of *p* < 0.5 and in bold italics *p* < 0.01. Here, DST = distal skin temperature (°C); LE = light exposure (log lux); ACCEL = acceleration (∑g/30 s); Tmov = movement time (counts/min); IS = interdaily stability; IV = intradaily variability; CFI = circadian function index; MIDDAY = mean of 10 consecutive hours with lowest values; TMIDDAY = timing for MIDDAY; and MEAN = mean value.

Controlled partial correlations for age and BMI are shown in Figure 5.

As was the case with the presence or absence of encephalopathy, of all the biochemical and hematological parameters analyzed, those related to the red series (red blood cell count, hemoglobin, and hematocrit) showed a stronger relationship with circadian parameters, especially with acceleration, time in movement, and light exposure (Figure 5).

Robustness (RA), CFI, midday, and 24 h mean values of acceleration and time in movement were all positively correlated with RBC, Hb, and Hto. In addition, regularity (IS) of time in movement was positively correlated with RBC, Hb, and Hto, whereas fragmentation (IV) was inversely correlated with these variables.

Regarding light exposure, IS, CFI, midday, and 24 h mean values were positively correlated with RBC, Hb, and Hto.

## 4. Discussion

Circadian parameters worsen with the severity of liver disease evaluated using the Child–Pugh score, which could be partly explained by the reduction in erythrocytes, hemoglobin, and hematocrit associated with the worsening of LC and the presence of encephalopathy. To the best of our knowledge, our results show, for the first time, the motor activity, light exposure, and distal skin temperature patterns of LC patients during a whole week in ambulatory conditions.

LC causes a variety of systemic disorders, from neuropsychiatric complications such as hepatic encephalopathy [5] to sleep problems, severe hemodynamic disturbances, and impaired metabolism, that can lead to malnutrition and sarcopenia.

All of these factors cause a serious loss in quality of life for the cirrhotic patient and sometimes require an intervention to improve the patient’s health, beyond their liver condition, to enable them to cope more adequately with a transplant, if one is prescribed.

Distal skin temperature is a reflection of the integrity of myogenic and neurogenic factors that regulate peripheral microvasculature and hence vasodilation in response to sympathetic–parasympathetic balance. Its rhythm is considered a good marker of the circadian system and is related to sleep quality, blood pressure, aging, metabolism, and neurodegeneration. A blunted rhythm was found to be related to the severity of chronic liver disease. Compared to the Child A group, Child C patients showed less stability, a flattening of the rhythm, due to an increase in daytime values, and a lower amplitude, as previously described for aging [27,34] or obstructive sleep disorder [35]. In a previous study which involved measuring distal and proximal skin temperature, cirrhotic patients showed higher distal skin temperatures during the daytime than healthy controls, which is consistent with our results [14].

Liver cirrhosis is associated with peripheral vasodilation and hyperdynamic circulation, which affects heat loss as a result of impaired autonomic control of the peripheral vasculature [19,36,37]. In addition to vasomotor alteration, there are other reasons why patients with cirrhosis may have an impaired temperature pattern, such as alterations in mitochondrial function [38,39] or systemic inflammation, associated with the severity of the disease.

Concerning physical activity, our results show that the worse the Child–Pugh score, the lower the quality and quantity of the movement. We used two methods for activity characterization, that is, acceleration and time in movement, because they are complementary in the information they provide about the patients. Unlike acceleration, which allows us to quantify the intensity–velocity of movements, time in movement does not distinguish between a slow and fast movement; it only measures the total time during which some type of movement is being performed, regardless of how fast or slow it is. Patients with a worse prognosis perform their physical activity less vigorously and more slowly. The relative inactivity of the more severe patients has been described previously as a characteristic of aged individuals [34], which is related to the aging process itself and an increased health risk [40]. In addition, the instability and fragmentation showed in the time in movement in severe patients have been previously described as indicators of mortality [41].

The causes of this reduced activity seem to be multiple. On one hand, the patients develop sarcopenia associated with the severity of the disease as a consequence of reduced muscular formation, together with impaired energy intake and malabsorption of macronutrients induced by biliary dysfunction and portal hypertension [3]. However, in 2013, Hayashi et al. found no differences in motor activity related to the Child–Pugh score, probably because most of their patients (86%) were scored as Child A [3]. On the other hand, the Child–Pugh scale takes into account the hepatic encephalopathy degree. It should be noted that from the early stages of hepatic encephalopathy, there may be apathy, progressing to incoordination and impaired movement, which leads to a progressive decrease in physical activity. Neuropsychiatric symptoms typically begin with subtle psychomotor changes [2]. To the best of our knowledge, this is the first time that the motor activity of patients with cirrhosis has been quantified. Our results show that from all of the recorded variables, motor activity, particularly time in movement, exhibited the greatest impairment associated with disease progression.

Light is considered the main zeitgeber for circadian system synchronization, and light therapy has been proposed to improve sleep quality in patients with cirrhosis. Regarding light exposure, our results show a progressive decrease in daytime light exposure and a tendency to increase nighttime light from Child A to Child C patients, as previously described for elderly people [34]. This quantity of light is not enough for circadian synchronizing [16].

Thus, the worsening of the circadian parameters associated with the severity of chronic liver disease could be generated by the disease process, which produces fewer active subjects, with more time indoors diminishing day–night contrast. Together, these could produce excessive daytime sleepiness and worse sleep quality, and thus, these patients could benefit from a brighter light and a physical activity program in the evening [16].

The patients included in this study showed alterations in hematological and biochemical analyses previously described in the literature, including alterations in sodium, HDLC, GGT, transferrin, RBD, Hb, Hto, PDW, and APTT [25,26,42,43,44]. Most of these are aggravated as the patient’s score worsens. It is true that, according to some authors, anemia in cirrhotic patients is an underestimated risk factor and should always be evaluated as an indicator of severity and even a predictor of transplant survival [45]. In this study, we have found a correlation between movement-related parameters, both in time and acceleration, and the red series. The correlation with RBC, Hb, and Hto is very interesting. Patients with greater robustness, 24 h mean and midday values, and stability in time in movement and acceleration have higher values for red cell-related parameters. Likewise, the higher the CFI (a CFI = 1 indicates an adequate circadian rhythm and a CFI = 0 indicates an absence of circadian rhythm), the higher the hematic parameters. However, we cannot establish whether the reduction in movement time and intensity results in a reduction in red blood cells and hemoglobin, or whether the reduction in red blood cells causes patients to refuse to move. Another element to consider is the fact that the Child–Pugh scale includes the presence or absence of encephalopathy, and it is very clear that Child C patients with encephalopathy are more likely to have a lower red blood cell count. When blood values are calculated depending on the presence of encephalopathy, there is an evident decrease in the red series in patients with encephalopathy, resulting in a reduction in red blood cell-related blood parameters. Skeletal muscle during exercise can produce erythropoietin, which is also able to stimulate erythropoiesis, as demonstrated in in vitro models [46]. A drastic reduction in the quantity and quality of daily exercise will reduce the possibility of erythropoietin production by skeletal muscle and could be an additional factor aggravating other factors that can lead to anemia in cirrhotic patients. The relationship between muscle mass and erythropoiesis has been demonstrated [47,48], and it has been found that patients on hemodialysis with sarcopenia show resistance to erythropoiesis-stimulating agents [49]. It has also been demonstrated that 12 weeks of moderate-to-vigorous intensity aerobic exercise significantly improved measures of cognition in individuals with HCV with suspected cognitive impairment [50].

Liver failure per se has also been reported to result in a reduction in red blood cell count, as bilirubin induces blood cell destruction [51], and portal hypertension resulting from cirrhosis present in many patients leads to splenic sequestration and red blood cell destruction [25]. Another factor to consider is that many of these patients have gastro-esophageal varices due to portal hypertension, which leads to bleeding and therefore a decrease in hematocrit and red blood cells, being a frequent cause of transfusion for gastrointestinal bleeding [52,53,54]. However, in this study, there was no correlation between the diagnosis of varices and the blood parameters. Different lines of action have been proposed to compensate for this effect, from iron and nutritional supplementation [55,56], to treatment with various drugs [45,55,56], to the most radical approaches, such as splenectomy with very fast responses, even 24–48 h after the intervention [57,58]. With these strategies, improvements in portal pressure, variceal bleeding, and cytopenia have been observed.

If we take all of the findings together and analyze them in a holistic way, we find that CH produces progressively with the severity of the disease a decrease in motor activity, both in quantity and quality, alterations in blood tests, as already demonstrated, a shift of the hours of light reception toward the night, and an alteration in the circadian rhythm of temperature. Defining this profile of alterations, especially findings such as temperature and movement alterations, will enable a holistic approach to patients with CH on the waiting list, as has already been proposed for other liver diseases [59]. The current reality in the hospital where this study was carried out, as in most hospitals, is that patients after liver transplantation receive holistic care based not only on medical attention but also on psycho-clinical support, nutritional care, and even sleep care. However, in the pretransplantation period, from inclusion in the waiting list, care is basically medical, without attending to the other points. Unless patients show a serious and evident alteration such as very marked sarcopenia or declare severe sleep disturbances, most of the care is focused on medical attention. Even in the pretransplant period, when holistic approaches to the management of CH are proposed, they are based on medical care. Thus, not only metabolic control but also mental disturbances, renal failure, cardiac dysfunction, and gastrointestinal bleeding are cited as action points [60]. Using tools such as actigraphs provides us with clinically relevant information that helps us understand the need to monitor not only the liver disease itself but also the consequences for other aspects of health that it may have.

Alterations in DST not only indicate physiometabolic alterations but could also be related to sleep, since during sleep, there is a variation in temperature of up to 1 °C. It has been shown that patients with CH often experience sleep disturbances, and more so for those with hepatic encephalopathy [61,62,63,64], although the latter is not consistent [62]. The most frequently cited causes for these disturbances are related to physical condition (fatigue), consequences of the disease (pruritus, medications, and pain), and poor sleep hygiene [65]. In another study, we found evident sleep disturbances in patients on the waiting list (unpublished data) which are probably reflected in the DST. Other evidence of sleep disturbance is increased exposure to light during the night and reduced exposure to sunlight as it worsens with the patient’s Child–Pugh score. The reasons may be diverse, from increased daytime sleepiness [62] to social isolation due to an inability to work. Moreover, this can become a vicious circle, since poor sleep hygiene will increase fatigue and hinder the development of a normal social and working life, not to mention the social stigma of LC. Almost 90% of patients in some studies have perceived some kind of stigmatization for their disease [66], which would further aggravate social isolation and increase the possibility of sleep disruption. Therefore, it seems clear that based on our results, both those shown here and those under analysis, there should be an outpatient diagnosis and intervention on sleep hygiene, if necessary.

Another area of action derived from our results is physical activity. It is obvious that physical activity is closely related to health. However, we are working with patients who show a progressive degradation in the quality of movement, both in the time and speed of movement, as demonstrated in this study. The worsening of the disease, and even the onset of hepatic encephalopathy, limits the movement capacity of patients. We cannot rule out that this progressive alteration in movement patterns is also related to the sleep disturbance and fatigue endured by the patients. In cirrhotic patients, a loss of muscle mass and contractile capacity has been identified to influence survival, quality of life, and even post-transplant outcomes [67,68], which can lead to a limitation on exercise, thus entering a vicious circle. Most of the information on the effect of physical exercise has been obtained in healthy individuals. There is also the effect it may have on cirrhotic patients, which must be carefully evaluated in terms of improvements in muscle mass, contraction, and oxygen consumption, but it is important to keep in mind that exercise may have an adverse effect on cirrhotic patients with elevated portal blood pressure [69]. However, endurance exercise of short duration is known to be safe in patients with LC, as opposed to resistance exercise, which could lead to aggravation of variceal bleeding and, given the limited knowledge on the effect derived from resistance exercise, should be avoided [69]. However, a 30 min daily program three to five times per week combining endurance and resistance exercises in a 3:2 ratio has also been proposed, and it has even been claimed that resistance exercises could be more effective in preventing sarcopenia [70]. The results of controlled moderate exercise programs lasting between 4 and 12 weeks are an improvement in muscle mass, peak activity capacity (VO_2_), muscle contractibility, portal pressure gradient, fatigue, and quality of life, suggesting that all patients with decompensated CH or not should receive an exercise prescription, obviously after adequate safety studies [71]. Moreover, it has even been suggested that since muscular force is an indicator of comorbidity and mortality, it should be routinely assessed in these patients, especially those who adhere to training or physical activity improvement programs [72]. Even very comprehensive patient safety assessment programs have already been proposed from different aspects, including the types of exercise patients could perform depending on their capabilities, which are worth exploring for implementation in patients on the waiting list [73].

Fatigue is another element that will limit the movement and quality of life of patients with cirrhosis. Numerous factors related to fatigue have been cited, but it has been documented that half of patients remain chronically fatigued one year after liver transplantation [74]. Patients’ fatigue should be assessed using at least self-perception questionnaires to determine the extent of the problem, since studies on primary biliary cirrhosis have shown a high correlation between self-perceived fatigue and the patient’s physical activity [75]. These data are not collected in any way by actuaries; therefore, in the holistic approach to the cirrhotic patient on the waiting list, an evaluation of fatigue by means of one of the questionnaires validated for this purpose should be included. Various therapies for liver disease fatigue have been explored and reviewed in a meta-study [76].

Nutrition also plays a critical role. It is obvious that nutrition and physical exercise are related. Therefore, exercise should be accompanied by adequate protein and caloric intake [77], or there is a risk of increasing patients’ actual or self-perceived fatigue. In patients with sarcopenia related to liver cirrhosis, variations in protein intake have been recommended, with a reduction in certain ammonia acids and the supplementation of others, focused on reducing the amount of ammonia [68], which can modify both sarcopenia and hepatic encephalopathy.

As for the clinical implications of the findings in this work, they are several. On the one hand, the use of these data can help us to improve the estimation of morbidity and mortality in cirrhotic patients on the waiting list, including, together with clinical and analytical data, the circadian rhythms of DST, ACCEL, Tmov, and light received. This would allow two lines of action to be established: By selecting the patients at the greatest risk, they could be prioritized on the waiting list with objective data and not only on the basis of the timing of their inclusion on the waiting list. And on the other hand, as a summary of the above, they could be included in a pretransplant prehabilitation program to prevent their clinical situation from worsening or to improve it for those patients in a situation of marked clinical deterioration.

Furthermore, these data open the door to various research projects that are already being carried out: the use of circadian data as a prognostic tool for predicting the short- and long-term survival of the transplanted patient, as well as translational studies of gene expression related to alterations in circadian rhythms and in relation to transplant performance. Both studies are already underway within the framework of the CRONORGAN21 project.

The authors are aware of the limitations of this study, especially the sample size. Even so, it should be taken into account that the study population is not easy to recruit and that the two years of the COVID-19 pandemic have made it very difficult to carry out all of the studies with patients. This article offers preliminary data that will be complemented in the future by increasing the study population.

## 5. Conclusions

Circadian system impairment, particularly in motor activity rhythms, is observed in patients with chronic liver disease, and it is associated with the progression and severity of the disease evaluated using the Child–Pugh score and is correlated with red blood parameters. These results already indicate the usefulness of circadian parameters to complement the clinical information provided by the Child–Pugh scale when considering transplant priority and establishing nutrition, physical exercise, and sleep guidelines that help ensure the better survival and quality of life of these patients.

## Figures and Tables

**Table 1:** Anthropometric characteristics of population sorted by Child–Pugh classification (mean ± SEM).

Child–Pugh	A		B		C		*p*-Value *
Sex	Male	Female	Male	Female	Male	Female	
N	14	4	22	7	10	6	
Age (yr)	59.5 ± 1.7	53.7 ± 5.7	58.2 ± 2.3	59.7 ± 2.3	54.4 ± 3.5	60 ± 3.8	0.660
Weight (Kg)	73.2 ± 3.6	65.9 ± 7.8	86 ± 4.5	62.9 ± 5.4	75.6 ± 4.1	67.5 ± 5.9	0.060
Height (cm)	167.9 ± 1.9	162 ± 0.7	173.1 ± 1.7	158.3 ± 2.4	172.8 ± 2.1	160 ± 2.9	0.401
IBM (Kgm^−2^)	25.9 ± 1.1	25.1 ± 3	28.6 ± 1.4	25.0 ± 1.8	25.2 ± 1.2	26.9 ± 1.9	0.209

* Comparisons have been made between 3 Child–Pugh score categories, taking all data and segmenting by sex.

**Table 2:** Non-parametrical indexes of circadian rhythms according to Child group (Child A, n = 18; Child B, n = 29; Child C, n = 16).

		IS	IV	RA	CFI	MIDDAY	TMIDDAY	MIDNIGHT	TMIDNIGHT	MEAN
ACCEL	Child A	0.31 ± 0.02	0.35 ± 0.03	0.73 ± 0.04	0.63 ± 0.02	17.46 ± 1.32	14:38 ± 00:23	2.48 ± 0.23	04:09 ± 00:19	11.14 ± 0.78
	Child B	0.30 ± 0.02	0.39 ± 0.02	0.58 ± 0.03 *	0.57 ± 0.02 *	11.51 ± 1.07 *	14:24 ± 00:19	2.82 ± 0.18	04:10 ± 00:16	7.30 ± 0.64 *
	Child C	0.27 ± 0.02	0.44 ± 0.03	0.51 ± 0.04 *	0.51 ± 0.02 *	9.09 ± 1.36 *	13:54 ± 00:24	2.75 ± 0.23	04:10 ± 00:21	6.77 ± 0.78 *
	*p*	0.223	0.059	0.001	0.000	0.000	0.419	0.481	0.999	0.000
Tmov	Child A	0.44 ± 0.03	0.26 ± 0.02	0.88 ± 0.03	0.73 ± 0.02	139.45 ± 8.19	14:24 ± 00:23	8.70 ± 1.61	03:32 ± 00:16	86.67 ± 5.27
	Child B	0.37 ± 0.02	0.33 ± 0.02 *	0.81 ± 0.03	0.67 ± 0.02	110.05 ± 6.72 *	14:38 ± 00:18	11.06 ± 1.30	04:11 ± 00:13	67.77 ± 4.41 *
	Child C	0.32 ± 0.03 *	0.37 ± 0.02 *	0.69 ± 0.03 *^#^	0.60 ± 0.02 *^#^	98.26 ± 9.05 *	14:32 ± 00:24	13.99 ± 1.66	04:03 ± 00:17	60.67 ± 5.64 *
	*p*	0.008	0.003	0.001	0.000	0.003	0.886	0.081	0.154	0.003
DST	Child A	0.51 ± 0.04	0.02 ± 0.00	0.04 ± 0.01	0.52 ± 0.02	31.48 ± 0.33	15:09 ± 00:50	34.23 ± 0.18	03:03 ± 00:43	32.68 ± 0.21
	Child B	0.50 ± 0.03	0.02 ± 0.00	0.04 ± 0.00	0.51 ± 0.01	31.76 ± 0.26	15:35 ± 00:42	34.45 ± 0.15	04:04 ± 00:35	32.87 ± 0.17
	Child C	0.36 ± 0.04 *^#^	0.03 ± 0.00	0.03 ± 0.01	0.46 ± 0.02	32.56 ± 0.34	15:27 ± 00:50	34.44 ± 0.19	02:05 ± 00:44	33.40 ± 0.22
	*p*	0.025	0.141	0.037	0.045	0.064	0.920	0.591	0.116	0.056
LE	Child A	0.61 ± 0.03	0.07 ± 0.01	0.97 ± 0.02	0.85 ± 0.01	1.76 ± 0.10	13:57 ± 00:22	0.01 ± 0.02	03:50 ± 00:13	0.97 ± 0.07
	Child B	0.58 ± 0.03	0.08 ± 0.01	0.95 ± 0.02	0.84 ± 0.01	1.56 ± 0.08	14:00 ± 00:18	0.05 ± 0.02	03:55 ± 00:11	0.89 ± 0.05
	Child C	0.47 ± 0.03 *^#^	0.07 ± 0.01	0.92 ± 0.02	0.78 ± 0.01 *^#^	1.22 ± 0.10 *^#^	15:03 ± 00:22	0.05 ± 0.02	04:27 ± 00:14	0.72 ± 0.07 *
	*p*	0.009	0.854	0.140	0.002	0.002	0.053	0.154	0.129	0.022

The main characteristics of the circadian rhythms studied: distal skin temperature, DST (°C); light exposure, LE (log lux); acceleration, ACCEL (∑g/30 s); and movement time, Tmov (counts/min) for Child A, Child B, and Child C patients. Interdaily stability (IS), intradaily variability (IV), relative amplitude (RA), circadian function index (CFI), the mean of the 10 consecutive hours with the lowest values for DST and the 10 consecutive hours with the highest values for LE, A, and MT (MIDDAY) with their corresponding timing (TMIDDAY), and the 5 consecutive hours with the highest values for the DST and the 5 consecutive hours with the lowest values for LE, A, and MT (MIDNIGHT) with their respective timing (TMIDNIGHT) and mean value (MEAN). IS, IV, RA, and CFI have no units, MIDDAY, MIDNIGHT, and MEAN are expressed in the units of their respective variable (DST in °C, LE in log10 lux, A in g and MT in counts/minute), while TMIDDAY and TMIDNIGHT are expressed in hours (hh:mm). Values are expressed as the mean ± SEM.**^#^** indicates statistically significant differences with the Child B group while * indicates statistically significant differences with Child A, according to the general linear model controlling for age, gender, and body mass index.

**Table 3:** Biochemistry results, sorted by Child–Pugh score. Data are shown as mean ± SEM.

Parameters\Child	Child A	Child B	Child C	*p*-Value	Normal Rank
Glu (mg/dL)	127.5 ± 10.4	138.1 ± 16.3	125.1 ± 5.5	NS	74–106
Ure (mg/dL)	41.5 ± 4.4	38.8 ± 5.1	47.3 ± 6.3	NS	17–48
Sod (mEq/L)	141.1 ± 0.9 ^a^	137.1 ± 0.9 ^b^	136.5 ± 1.4 ^b^	0.01	136–145
Crea (mg/dL)	0.8 ± 0.1	1.2 ± 0.3	1.1 ± 0.2	NS	0.7–1.2
Pprot (g/dL)	6.9 ± 0.3	7.2 ± 0.2	6.7 ± 0.1	NS	6.4–8.3
Alb (g/dL)	5.1 ± 1.0	3.6 ± 0.1	3.5 ± 0.2	NS	3.5–5.2
Tbil (mg/dL)	1.1 ± 0.3	1.8 ± 0.3	2.7 ± 0.7	0.077	0.05–1.2
DirBil (mg/dL)	2.0 ± 0.6	1.3 ± 0.3	2.1 ± 0.8	NS	<0.3
AFP (U/L)	4.1 ± 0.7	55.0 ± 51.6	3.5 ± 0.9	NS	20–130
Cholin (U/L)	5451.0 ± 1010.0	3738.0 ± 466.0	2519.2 ± 853.0	0.061	8000–18000
Chol (mg/dL)	165.2 ± 12.1	154.1 ± 8.9	122.3 ± 19.0	NS	<190
TG (mg/dL)	131.3 ± 20.3	95.2 ± 7.1	98.0 ± 22.6	NS	<150
HDLC (mg/dL)	62.6 ± 7.7 ^a^	49.3 ± 4.9 ^ab^	35.6 ± 5.1 ^b^	0.027	>46
LDLC (mg/dL)	75.5 ± 15.5	104.0 ± 11.2	76.6 ± 17.9	NS	<70
GOT-AST (U/L)	60.8 ± 7.3	47.4 ± 7.6	58.2 ± 9.7	NS	5–40
GPT-ALT (U/L)	70.1 ± 12.7	39.6 ± 8.8	38.7 ± 8.5	0.067	5–41
ALP (U/L)	171.1 ± 26.4	168.3 ± 18.6	212.9 ± 37.3	NS	40–130
GGT (U/L)	215.6 ± 44.5 ^a^	99.9 ± 15.8 ^b^	63.6 ± 16.7 ^b^	0.001	10–71
Trans (mg/L)	299.8 ± 46.6 ^a^	226.4 ± 18.4 ^ab^	181.3 ± 28.9 ^b^	0.053	200–360
Ferr (ng/mL)	113.8 ± 55.4	293.6 ± 82.8	252.6 ± 93.9	NS	30–400

Here, Glu = glucose, Ure = urea, Sod = sodium, Crea = creatinine, Pprot = plasma proteins, Alb = albumin, TBil = total bilirubin, DirBil = direct bilirubin, AFP = alpha-fetoprotein, Cholin = cholinesterase, Chol = cholesterol, TG = triglyceride, HDLC = high-density lipoprotein cholesterol, LDLC = low-density lipoprotein cholesterol, GOT-AST = glutamic oxaloacetic transaminase or aspartate aminotransferase, GPT-ALT = glutamic pyruvic transaminase or alanine aminotransferase, ALP = alkaline phosphatase, GGT = gamma-glutamyl transpeptidase, Trans = transferrin, and Ferr = ferritin. Different superscripts in the same line means significant differences.

**Table 4:** Hematological values sorted by Child–Pugh score. Data are shown as mean ± SEM.

Parameters\Child	Child A	Child B	Child C	*p*-Value	Normal Rank
RBC (10^6^/µL)	4.4 ± 0.2 ^a^	3.8 ± 0.1 ^b^	3.4 ± 0.2 ^b^	0.001	4.5–5.9
Hb (g/dL)	12.6 ± 0.7 ^a^	11.5 ± 0.4 ^ab^	9.9 ± 0.4 ^b^	0.004	13.5–17.5
Hto (%)	37.2 ± 2.2 ^a^	33.7 ± 1.1 ^ab^	29.9 ± 1.2 ^b^	0.009	41–53
MCV (fL)	86.3 ± 3.9	87.3 ± 3.6	90.2 ± 2.3	NS	80–100
MCH (pg/cell)	33.0 ± 2.9	30.9 ± 0.7	30.4 ± 1.1	NS	26–34
MHCH (g/dL)	33.1 ± 0.6	34.1 ± 0.3	33.2 ± 0.3	NS	31–36
RDW (%)	15.9 ± 1.1	15.2 ± 0.4	16.6 ± 0.6	NS	11.5–14.5
PLT (10^3^/µL)	101.4 ± 14.6	101.0 ± 10.4	115.8 ± 13.4	NS	150–350
MPV (fL)	11.7 ± 0.3	10.9 ± 0.2	10.9 ± 0.4	0.086	6.4–11.0
PDW (%)	15.9 ± 0.8 ^a^	13.1 ± 0.5 ^ab^	14.3 ± 0.9 ^b^	0.017	9–17
WBC (10^3^/µL)	5.7 ± 0.5	5.1 ± 0.5	5.0 ± 0.5	NS	4.5–11.0
NEU (10^3^/µL)	3.6 ± 0.5	3.3 ± 0.5	3.2 ± 0.4	NS	1.8–7.7
LYMP (10^3^/µL)	1.4 ± 0.2	1.1 ± 0.1	1.0 ± 0.1	NS	1–4
MONO (10^3^/µL)	0.5 ± 0.0	0.5 ± 0.1	0.5 ± 0.1	NS	0.0–0.8
EOSI (10^3^/µL)	0.1 ± 0.0	0.2 ± 0.0	0.2 ± 0.0	NS	0.0–0.5
BASO (10^3^/µL)	0.0 ± 0.0	0.0 ± 0.0	0.1 ± 0.1	NS	0.0–0.2
FIBRI (mg/dL)	383.5 ± 24.3	294.5 ± 30.3	338.1 ± 35.5	NS	276–471
PTT (s)	12.9 ± 0.3	17.5 ± 1.6	17.6 ± 0.8	0.069	9.4–12.5
APTT (s)	83.9 ± 2.9 ^a^	65.5 ± 4.2 ^b^	61.9 ± 4.6 ^b^	0.020	25.1–36.5
INR	1.2 ± 0.0	1.5 ± 0.1	1.6 ± 0.2	0.073	0.9–1.2
TTP (s)	34.4 ± 1.3	38.5 ± 1.8	37.6 ± 1.4	NS	20–40
APTTR (s)	1.2 ± 0.0	1.4 ± 0.1	1.2 ± 0.0	0.081	<1.3

Here, RBC = red blood cell count, Hb = hemoglobin, Hto = hematocrit, MCV = mean corpuscular volume, MCH = mean corpuscular hemoglobin, MCHC = mean corpuscular hemoglobin concentration, RDW = red cell distribution width, PLT = platelet count, MPV = mean platelet volume, PDW = platelet distribution width, WBC = white blood cell count, NEU = neutrophil, LYMP = lymphocyte, MONO = monocyte, EOSI = eosinophil, BASO = basophile, FIBRI = fibrinogen, PTT= partial prothrombin time, APTT = activated partial thromboplastin time, INR = international normalized ratio, TTP = activated partial thromboplastin time, and APTTR = activated partial thromboplastin time ratio. Different superscript in the same line means significant differences.

**Table 5:** RBC, Hb, Hto, and PLT depending on the detection of hepatic encephalopathy at the time of recruitment.

Parameter	Encephalopathy		*p*-Value
	NO	YES	
RBC (10^6^/µL)	4.07 ± 0.16	3.57 ± 0.11	0.014
Hb (mg/dL)	12.15 ± 0.43	10.74 ± 0.43	0.03
Hto (%)	35.95 ± 1.26	31.9 ± 1.12	0.028
PLT (10^3^/µL)	115.85 ± 9.36	85.11 ± 9.4	0.03

Here, RBC = red blood cell count, Hb = hemoglobin, Hto = hematocrit, and PLT = platelet count.

## Data Availability

The data cannot be shared due to confidentiality protection of the patients.
